# Limited ankle dorsiflexion increases the risk for mid-portion Achilles tendinopathy in infantry recruits: a prospective cohort study

**DOI:** 10.1186/s13047-014-0048-3

**Published:** 2014-11-18

**Authors:** Alon Rabin, Zvi Kozol, Aharon S Finestone

**Affiliations:** Department of Physiotherapy, Ariel University, Ariel, Israel; Department of Orthopaedic Surgery, Assaf Harofeh Medical Center, Zerrifin, Israel; Sackler School of Medicine, Tel-Aviv University, Tel-Aviv, Israel; Israel Defense Force Medical Corps, Haifa, Israel

**Keywords:** Achilles, Tendinopathy, Risk, Ankle, Dorsiflexion

## Abstract

**Background:**

Achilles tendinopathy (AT) is a prevalent condition among runners and military personnel. Although ankle dorsiflexion (DF) range of motion (ROM) as measured with the knee bent has not been previously associated with AT, the literature concerning its role is limited. In addition, the role of lower extremity movement pattern in the pathogenesis of AT has not been studied prospectively.

The purpose of this study was to further explore the role of ankle DF ROM as measured with the knee bent and that of lower extremity movement pattern as risk factors for mid-portion AT.

**Methods:**

Seventy healthy male military recruits (mean ± SD age, height and body mass of 19.6 ± 1.0 years, 176.0 ± 10.0 cm, and 71.5 ± 7.4 kg) participated in this study. Ankle DF ROM as measured with the knee bent in weight-bearing (WB) and non-weight-bearing (NWB), as well as lower extremity quality of movement during a lateral step down (LSD) test were measured at baseline. Participants were then followed for a 6-month period of army basic training with recording of the development of AT.

**Results:**

Five participants developed AT during training. Participants that developed AT had a more limited NWB ankle DF ROM (27.4^0^ versus 21.1^0^, *p* = 0.025). The quality of lower extremity movement did not differ between injured and uninjured participants (*p* = 0.361).

**Conclusions:**

A more limited ankle DF ROM as measured in NWB with the knee bent increases the risk of developing AT among military recruits taking part in intensive physical training.

## Background

Achilles tendinopathy (AT) is a common injury among runners and military personnel [1-7]. The disorder is typically classified as insertional, affecting the tendon at its insertion onto the calcaneus, or as mid-portion, affecting the tendon between 2 to 6 cm proximal to its insertion [8]. This paper will focus on the latter of these 2 entities.

Despite its frequency, only few prospective investigations into the risk factors for AT have been performed. Risk factors are typically classified as intrinsic, or extrinsic. Intrinsic risk factors that have been previously associated with AT include increased age, increased or decreased gastrocnemius flexibility, decreased subtalar motion, increased pronation, and decreased plantar flexor strength [8,9].

Although the flexibility of the gastrocnemius has been previously associated with AT [10,11], the kinematics of running suggest it is the flexibility of the soleus that may be more relevant to the pathogenesis of this condition. While flexibility of the gastrocnemius is assessed by measuring ankle dorsiflexion (DF) with the knee extended, this alignment is never reached during running [12]. Instead, maximal ankle DF is reached during the mid-stance of the running gait cycle, when the knee is flexed approximately 40^0^ [12,13]. The simultaneous ankle DF and knee flexion that occur during the first half of the running cycle, are controlled eccentrically by the ankle plantarflexors and serve to absorb the impact of the upper body [12,14]. This shock-absorbing mechanism is likely to strain the soleus more than the gastrocnemius as its origin on the tibia makes it suitable for controlling knee flexion under weight-bearing conditions. In fact, it has been previously shown that during running the contribution of the soleus to power absorption increases as compared with walking [15]. Therefore, a measurement of ankle DF with the knee bent, which reflects soleus flexibility, may be more indicative of the functional demands during running. Furthermore, as it has been shown that a more limited flexibility of the gastrocnemius increases absorption work by the plantarflexors during walking [16], it is possible, that a more limited flexibility of the soleus would likewise increase absorption work by the plantarflexors during running, which may lead to increased strain on the Achilles tendon.

Although when previously tested, bent-knee ankle DF has not been associated with AT, findings are limited by few prospective studies [10,11]. Therefore, further investigation of this association seems warranted.

Another possible risk factor for AT is an altered lower extremity movement pattern [17]. AT has been associated with excessive foot pronation during running [8], as well as greater hip adduction and knee internal rotation during a leaping maneuver [17]. These kinematic alterations are part of a more general movement pattern involving a medial collapse of the knee which is sometimes referred to as “dynamic knee valgus” [18,19]. Dynamic knee valgus which has been associated with several other lower extremity pathologies [20,21], may also increase the risk for AT.

The current gold standard for assessing lower extremity movement pattern is 3-dimensional motion analysis. However, due to its cost, unique set-up, length of application and required training, this tool may not be feasible in the clinical setting. If a more clinically feasible assessment tool can successfully identify individuals at risk for developing AT this may constitute significant progress in risk assessment, and possibly in injury prevention. The lateral step down (LSD) is a reliable test often used in clinical practice to assess lower extremity movement pattern [22-24]. However, the predictive validity of the LSD in determining the risk for AT, has yet to be determined.

The purpose of this study was to examine whether ankle DF as measured with the knee bent, as well as the quality of movement as measured by the LSD can predict the development of AT in male military recruits undergoing army basic training (ABT).

## Methods

The study was approved by the Institutional Review Board of the Israel Defense Forces (approval No. IDF 964–2010), and all participants provided informed consent prior to participation.

### Participants

Seventy male participants with a mean ± SD age 19.6 ± 1.0 years, height 176.0 ± 10.0 cm, and body mass 71.5 ± 7.4 kg were recruited for this study. Participants were military recruits who were thoroughly screened for any musculoskeletal injury/condition prior to beginning a 26-week ABT period. Inclusion criteria were age 18 years or older and no current complaint of pain in the lower extremities or lumbar spine. Participants were excluded if they could not perform any of the measurements included in the study due to pain, imbalance, or any other limitation.

### Examiners

Four examiners performed data collection for this investigation. Two examiners were physical therapists. One physical therapist, who had 15 years of clinical experience in the management of musculoskeletal conditions, performed all DF ROM measurements. The other physical therapist, with over 25 years of teaching and clinical experience in the field of kinesiology and neurological rehabilitation, performed all LSD assessments. These physical therapists have previously demonstrated a moderate inter-rater reliability when performing the LSD (kappa 0.59) [24], and an excellent inter-rater reliability when performing the weight-bearing (WB) and non-weight-bearing (NWB) DF ROM measurements (intraclass correlation coefficient 0.95 and 0.86, respectively) [24]. Prior to data collection, the physical therapists met for a 4-hour session in order to review each measurement procedure. The 2 other examiners were orthopaedic surgeons. Both surgeons had more than 15 years of experience in foot and ankle surgery among training populations and more than 15 years of experience in research of overuse injuries. The surgeons were responsible for determining the diagnosis of AT during the follow-up period based on pre-determined criteria.

### Baseline measurements

Demographic data including age, height, body mass, and any past Achilles tendon disorder were collected at baseline. Lower extremity quality of movement (LSD test) and ankle DF ROM were subsequently measured. Ankle DF was measured in WB and NWB, as it has been previously suggested that these measurements may be assessing 2 different constructs [25]. All measurements were performed bilaterally.

**Figure 1:**
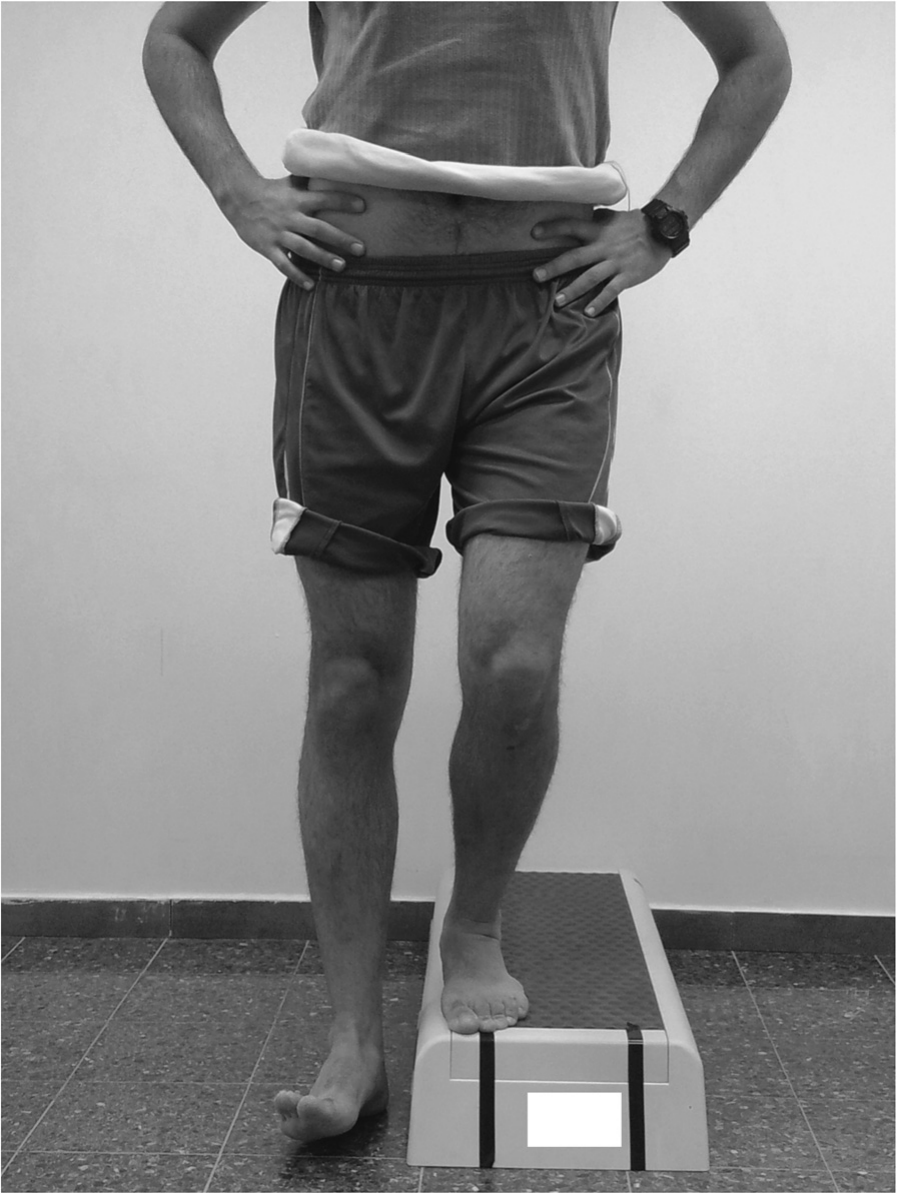
**Lateral step down test – good quality of movement.**

The LSD test was performed on a 15 cm step (Reebok International, Canton, MA, USA). Participants stood by the edge of the step. They were instructed to keep their trunk straight, hands on their waist, and bend their knee until the contralateral heel touched the floor next to the step. Participants were asked not to put any weight on the heel once it reached the floor. Participants were also asked to try to maintain the knee of the tested leg over the 2nd toe of the ipsilateral foot during the test (a perpendicular black tape was placed along the front of the step from just under the participant’s 2nd toe to the floor in order facilitate the assessment by the examiner) (Figure 1). Participants were given 5 practice repetitions before performing 5 consecutive test repetitions. The side tested first was alternated between consecutive participants.

**Figure 2:**
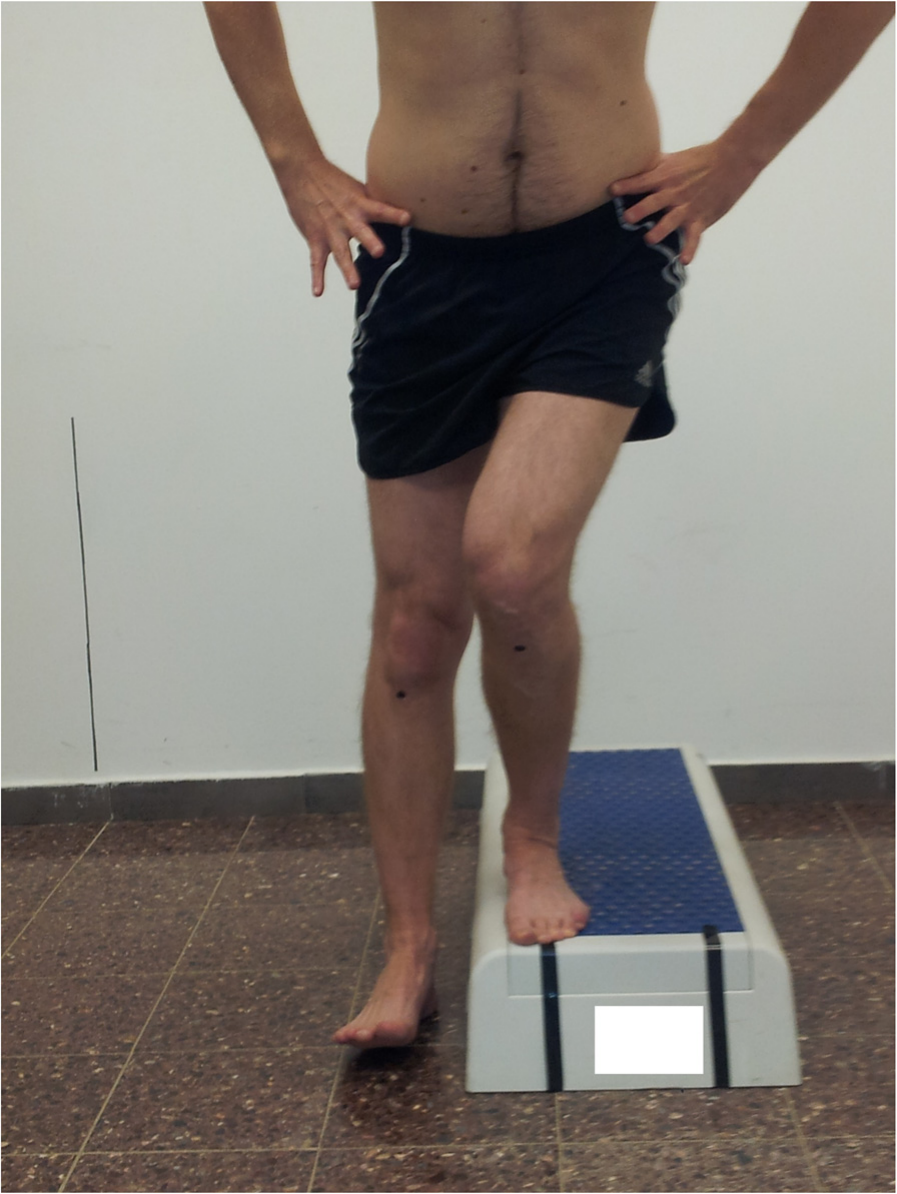
**Lateral step down test – moderate quality of movement.**

The examiner was positioned 3 meters in front of the participant and scored the test based on a 7-point scale (0 to 6) [23] (Table 1). If any one of the movement deviations outlined in Table 1 was observed during any of the repetitions, the participant was considered as having demonstrated that deviation, and the corresponding point value was assigned. A total score of 0 to 1 was classified as “Good” quality of movement (Figure 1), a total score of 2 to 3 was classified as “Moderate” quality of movement (Figure 2), and a total score of 4 or more was classified as “Poor” quality of movement [23]. As only 3 participants demonstrated a “Poor” quality of movement they were grouped together with participants demonstrating a “Moderate” quality of movement for data analysis.

**Table 1:** **Scoring criteria for the lateral step down test**

**Criterion**	**Interpretation**	**Score**
**Arm strategy**	Removal of a hand off the waist	1
**Trunk alignment**	Leaning in any direction	1
**Pelvis plane**	Loss of horizontal plane	1
**Knee position**	Tibial tuberosity medial to 2nd toe	1
	Tibial tuberosity medial to medial border of foot	2
**Steady stance**	Participant stepped down on non tested limb, or the foot wavered from side to side	1

For the WB DF ROM measurement a 50 cm long line was marked on the floor and a continuous 60 cm long line was marked on a wall where the test was to be performed. The participant placed the tested foot along the floor line so that the line bisected the heel and the second toe was on the line. The participant was then asked to lunge forward and bring his patella as close as possible to the vertical line drawn on the wall without lifting the heel off the floor. Once maximal DF was reached the examiner placed an inclinometer (MIE Inclinometer, Nationwide Medical, Inc. Agoura Hills, CA, USA), which was first zeroed on a fixed vertical reference, over the anterior tibia 15 cm below the tibial tuberosity. The DF angle was recorded and the participant returned to the starting position. The average of 3 measurements was used for data analysis.

**Figure 3:**
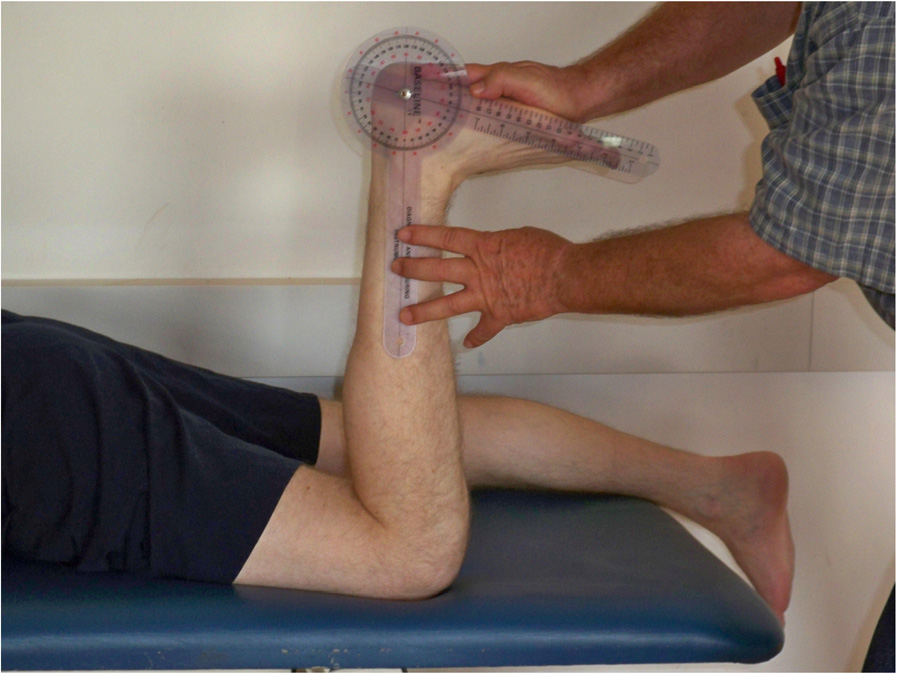
**Non-weight-bearing ankle dorsiflexion measurement.**

For the NWB DF ROM measurement the participant assumed a prone lying position with the knee bent 90^0^ (Figure 3). The measurement was taken using a universal goniometer (Baseline Plastic Goniometer, The Therapy Connection Inc., Windham, NH, USA). The examiner first manually verified a subtalar neutral position and then leaned his body weight forward over his hands in order to stretch the ankle fully into DF. Dorsiflexion was measured as the angle between the lateral midline of the lower leg (a line from the head of the fibula to the tip of the lateral malleolus) and the lateral border of the foot (a line along the rearfoot/calcaneus). The average of 3 measurements was used for data analysis.

The side tested first, as well as the order of the different DF measurements were alternated between consecutive participants.

### Army basic training period

Participants initiated a 26-week ABT period immediately following baseline measurements. During this period participants went through rigorous physical fitness, as well as military skill training, according to a set and progressively intensified schedule. All participants were exposed to the same training regimen and trained under similar conditions in terms of shoe wear, loads carried, nutrition, and rest periods.

### Injury tracking system

The 2 orthopaedic surgeons met the participants once every 2–3 weeks throughout the course of training. Any complaint was assessed by one of the surgeons with a physical examination, however, no imaging studies were used to verify the diagnosis of AT in this study. A participant was considered as having AT if he complained of pain over the tendon during physical activity and presented with tenderness to palpation over the tendon in the area between 2 to 6 cm proximal to the calcaneal insertion. The participant had to report that palpation over this area reproduced his symptoms. Additional symptoms and signs such as morning stiffness, swelling, warmth, or redness were sought as well, however they were not considered mandatory for establishing the diagnosis.

### Statistical analysis

Baseline measurements were summarized with descriptive statistics using measures of central tendency and dispersion for continuous variables and frequency counts for categorical variables. Univariate associations between the outcome and potential risk factors were analyzed using Mann–Whitney and Fisher Exact tests for interval and categorical variables, respectively. Any variable demonstrating a significant association (*p* < 0.05) was entered into a univariate logistic regression model in order to determine its predictive power. Continuous variables that were significantly associated with the outcome were further analyzed using receiver-operator characteristic (ROC) curves in order to establish their best cutoff value. This cutoff value, which was identified visually as the point nearest the upper left-hand corner of the diagram, corresponds with maximal sensitivity and specificity. All analyses were performed using JMP version 10 statistical package (SAS Institute, Cary, NC) with an a-priori level of significance of *p* < 0.05.

## Results

Seventy-five participants were screened. One participant declined participation; another participant was excluded due to suffering an ankle sprain the day before baseline testing, and 2 participants were expelled from ABT for reasons unrelated to their health. A fifth participant died during the follow-up period from septic shock. Thus 70 participants were included in the final analysis. Table 2 presents demographic, history, and physical examination variables of the entire sample, as well as the differences between participants that developed AT and those that did not.

**Table 2:** **Demographic, history and clinical variables among the sample**

**Variable**	**All participants (N = 70)**	**Injured (N = 5)**	**Non-injured (N = 65)**	***P*** **-value**
**Age, y** ^**a**^	19.6 ± 1.0	19.7 ± 0.8	19.6 ± 1.0	0.661
**BMI** ^**a**^	23.1 ± 2.1	23.2 ± 2.8	23.1 ± 2.0	0.965
**Past AT, n (%) yes** ^**b**^	0 (0.0)	0 (0.0)	0 (0.0)	NA
**Right WB DF,** ^**0 a**^	49.4 ± 6.7	43.3 ± 9.1	49.9 ± 6.3	0.184
**Left WB DF,** ^**0 a**^	56.4 ± 6.8	50.2 ± 5.8	56.8 ± 6.7	0.025
**Right NWB DF,** ^**0 a**^	27.4 ± 5.9	21.1 ± 6.1	27.9 ± 5.6	0.025
**Left NWB DF,** ^**0 a**^	27.3 ± 6.3	21.3 ± 7.3	27.8 ± 6.0	0.067
**Right LSD, n (%) good (score 0 to 1)**	33 (47.1)	1 (20.0)	32 (49.2)	0.361
**Left LSD, n (%) good (score 0 to 1)**	33 (47.1)	1 (20.0)	32 (49.2)	0.361

^a^Values are Average ± SD.

^b^No statistics are computed for this parameter as it is constant.

*Abbreviations: AT* Achilles Tendinopathy, *BMI* Body Mass Index, *DF* Dorsiflexion, *LSD* Lateral Step Down, *NWB* Non-Weight-Bearing, *WB* Weight-Bearing.

Overall, 5 participants were diagnosed with AT during ABT. All injuries occurred in the right leg. Ankle DF as measured in NWB was more limited among participants that developed AT (*p* = 0.025). Univariate logistic regression also indicated that NWB ankle DF ROM significantly predicted AT (*p* = 0.007). The unit odds ratio (OR) for NWB DF in predicting AT was 0.77 (95% CI 0.59 – 0.94) which indicates, that for every 1-degree of increased NWB DF ROM, the odds of developing a future AT reduced by 0.23. The ROC curve for NWB DF ROM indicated that the best cutoff value for determining the risk of AT was 22^0^, with a corresponding sensitivity and specificity of 80% and 86%, respectively, and an area under the curve of 0.80.

No difference in quality of movement (LSD) was noted between healthy participants and those with AT (*p* = 0.361).

## Discussion

Ankle DF ROM measured with the knee bent was predictive of the development of AT in a sample of 70 military recruits. To the best of our knowledge, this study is the first to implicate this factor in the risk of AT.

The difference between uninjured participants and those that developed AT exceeded the minimal detectable change of the DF measurement that was used in this study (6.2^0^) [25], thus further supporting the existence of a true DF ROM difference between these 2 groups. The most accurate DF cutoff threshold for predicting AT was 22^0^, with 4 of the 5 injured participants demonstrating range values below this threshold, compared to only 9 of 65 uninjured participants. Previous investigations suggest that the ankle dorsiflexes approximately 20^0^ during the stance phase of running [13,26]. This means that if the available DF ROM of an athlete was approximately 20^0^, their ankle would need to be stressed very close to its DF limit with every step during running. Over time, this may lead to injury.

Although its role in the pathogenesis of AT is controversial [9], subtalar pronation may be another mechanism by which limited ankle DF may cause AT. A more limited DF ROM may lead to compensatory subtalar pronation [27] which, in turn, may lead to greater tibial internal rotation. When this occurs during mid- to late-stance it may cause “wringing out” of the Achilles tendon due to the simultaneous knee extension which is accompanied by tibial external rotation [8,14]. Excessive subtalar pronation may also contribute to the development of AT as it necessitates greater activation of the gastro-soleus complex during running [8].

Unlike NWB DF ROM, the WB measurement did not reveal a significant difference between injured and uninjured participants. Although injured participants did display 6^0^ less WB DF, this difference was mainly due to extremely low values among 2 of the 5 injured participants (31.1^0^ and 35.7^0^), while the other 3 participants displayed range values that were very close to the sample mean (48.3^0^, 50.0^0^ and 51.0^0^ degrees). The discordance between WB and NWB DF is not surprising given that either measurement has been shown to account for only 35% of the variance in the other [25]. Interestingly WB DF ROM on the left side was more limited among injured compared with uninjured participants (Table 2), and a similar trend was noted for left NWB DF ROM. Combined with the findings on the right side, these differences suggest that injured participants tended to display a more limited ankle DF ROM bilaterally. The fact that all injuries still occurred on the right side may be related to differences in the usage of the 2 extremities. For example, it has been previously shown that during gait the power generation of the right lower extremity is more associated with propulsion, while that of the left is more associated with postural control [28]. Consequently, even in the presence of a symmetrically limited DF ROM, the right side may still be more prone to developing AT. The association between sidedness and the development of AT, as well as other overuse lower extremity injuries, may be worth exploring.

Although not statistically significant, a greater proportion of injured participants displayed a faulty lower extremity movement pattern during the LSD test (80% among injured participants versus 51% among uninjured participants). This movement pattern has been previously associated with decreased ankle DF ROM and increased subtalar pronation [24,29]. Three of the injured participants displayed a moderate movement pattern (score 3/6) during the LSD test, while a fourth participant, which was subsequently grouped with the “Moderate” quality group, actually displayed a “Poor” pattern based on the LSD scoring criteria (score 4/6). Due to the overall small number of injured participants, the possibility of a type 2 error should be considered, and we recommend further testing of the predictive validity of the LSD test in AT. Furthermore, it is possible that with the use of a more quantitative assessment method of joint and/or segment alignment during the LSD, the predictive ability of this test would be enhanced.

Our findings are at odds with 2 previous studies that did not find an association between limited bent-knee ankle DF and AT [10,11]. These differences may be partly explained by the way DF was measured. The DF ROM in our study was somewhat larger than that found by these studies (27^0^ in our study vs. 20-23^0^ previously) [10,11]. Mahieu et al. [11] measured DF both actively and passively in a supine (gravity-resisted) position, with the knee bent 45^0^ [11]. We used a prone (gravity-assisted) position with the knee bent 90^0^, which may explain the greater ROM found in our study. Although measuring ankle DF ROM with the knee bent approximately 40^0^ may be more representative of the functional demands during running, we believe the prone 90^0^ knee flexion position, which places the tibia in a vertical alignment, allows the examiner to use his/her body weight more effectively in order to stretch the ankle fully into its DF limit. In the study by Kaufman et al. [10] neither the position of the measurement, nor whether active or passive motion was measured, were specified [10]. Finally, while no information regarding measurement reliability was provided by these previous investigations, our technique has been previously proven reliable when performed by the examiners involved in this study [24].

Our study has several important limitations. First, the number of cases of AT was low (n = 5), leading to a wide 95% CI around the point value of the predictor. The incidence of AT in our study was 7%, which is similar to that found by Kaufman et al. [10] over a similar follow-up period (5%), but lower than that found by Mahieu et al. [11] over a 6-week follow-up period (14%). As the diagnosis of AT was based on history and physical examination in all studies, we believe differences in training regimens, rather than diagnostic criteria, are more likely to explain the different incidence rates. The lack of use of imaging for the diagnosis of AT could be considered another limitation of this study. However, our diagnostic criteria are compatible with current clinical practice guidelines [8]. Furthermore, recent evidence suggests that pain on palpation of the tendon, and the subjective report of pain between 2 to 6 cm proximal to the insertion of the tendon, are the most accurate diagnostic criteria for AT [30]. Our findings may also be limited to a relatively young population, undergoing rigorous physical training. Nutritional regimen, hours of sleep, training terrains, loads carried and shoe wear may also differ between a military and a civilian population, thus presenting another possible limitation. Finally, due to a limited number of investigators and participants, we were not able to obtain other possible predictors during baseline testing. Thus, the effects of other variables such as DF ROM with the knee extended, subtalar mobility, endurance or strength of various muscle groups could not be considered.

## Conclusions

Limited ankle DF ROM, as measured in NWB with the knee bent, may increase the risk of developing AT in army recruits taking part in intense physical training. Future studies are needed to further validate limited bent-knee ankle DF as a risk factor for AT, as well as to assess whether a cutoff threshold of 22^0^ can most accurately predict the occurrence of this condition. Finally, despite its inability to predict AT in this study, we encourage future investigation of the LSD test as a screening tool for the risk of AT.

## Abbreviations

ABT: Army basic training
AT: Achilles tendinopathy
DF: Dorsiflexion
LSD: Lateral step down
NWB: Non-weight-bearing
OR: Odds ratio
ROC: Receiver operator curve
ROM: Range of motion
WB: Weight-bearing
